# Current Strategies for Identification of Glioma Stem Cells: Adequate or Unsatisfactory?

**DOI:** 10.1155/2012/376894

**Published:** 2012-05-23

**Authors:** Paola Brescia, Cristina Richichi, Giuliana Pelicci

**Affiliations:** Department of Experimental Oncology, European Institute of Oncology (IEO), 20139 Milan, Italy

## Abstract

Cancer stem cells (CSCs) were isolated in multiple tumor types, including human glioblastomas, and although the presence of surface markers selectively expressed on CSCs can be used to isolate them, no marker/pattern of markers are sufficiently robust to definitively identify stem cells in tumors. Several markers were evaluated for their prognostic value with promising early results, however none of them was proven to be clinically useful in large-scale studies, leading to outstanding efforts to identify new markers. Given the heterogeneity of human glioblastomas further investigations are necessary to identify both cancer stem cell-specific markers and the molecular mechanisms sustaining the tumorigenic potential of these cells to develop tailored treatments. Markers for glioblastoma stem cells such as CD133, CD15, integrin-**α**6, L1CAM might be informative to identify these cells but cannot be conclusively linked to a stem cell phenotype. Overlap of expression, functional state and morphology of different subpopulations lead to carefully consider the techniques employed so far to isolate these cells. Due to a dearth of methods and markers reliably identifying the candidate cancer stem cells, the isolation/enrichment of cancer stem cells to be therapeutically targeted remains a major challenge.

## 1. Introduction

The cancer stem cell hypothesis postulates that a small subpopulation of cancer cells possessing self-renewal characteristics are responsible for initiating and maintaining cancer growth. According to the CSC model, the large populations found in a tumor might represent diverse stages of differentiation. The biological characteristics shared by normal stem cells (NSCs) and CSCs mainly involve self-renewal and differentiation potential, survival ability, niche-specific microenvironment requirements, and specific homing to injury sites and may have important implications in terms of new approaches to cancer. The identification of new therapeutic targets based on the CSC model represents a great challenge.

Glioblastoma multiforme (WHO grade IV) is the most aggressive among the brain tumors of adults and displays striking morphologic variation among different patients. GBM contains a mixture cell populations with high propensity to infiltrate throughout the brain (making complete surgical resection impossible). It has been demonstrated that the bulk of malignant cells in GBM is generated by rare fractions of self-renewing, multipotent tumor-initiating cells (CSCs) also called tumor-initiating cells or tumor-propagating cells [1, 2] responsible for tumor growth and recurrence and resistance to chemo- and radiotherapies [3].

Cancer stem cells generate tumors with the cardinal features of the GBMs from which they derived, including an infiltrative phenotype and histopathological features such as hypercellularity, pseudopalisading necrosis, and angiogenesis. With the available tools, it is difficult to isolate such cells directly from biopsies since the bias derived from the heterogeneity of the cellular composition of the specimen will not give reliable and precise results. The approach of processing and isolating the glioma stem cells from the original tumor gives rise to inconsistencies among various groups since it is not yet clear how stable is the phenotype of the glioma stem cell.

Although in some tumors, such as breast, prostate, pancreas, skin, colon, and blood cancer, the presence of a slowly cycling and highly tumorigenic cell fraction is recognized [4–12], no single antigen has been shown to reliably segregate tumorigenic stem cells from the rest of the tumor specimen.

The identification of tumoral neural stem cells will provide a powerful tool to investigate the tumorigenic process in the central nervous system and to develop therapies targeted to these cells. This takes on additional importance in the light of the demonstration that aberrant and multiple states of differentiation may be present within the same tumor. To date, current techniques have neither isolated nor defined profiles absolutely representative of a stem cell.

Although several markers may be informative towards brain tumor stem cell identification, the segregation of universal, specific markers that might be reliably used to distinguish a normal stem cell from a cancer stem cell as well as a stem cell from a progenitor is still inadequate.

There is an overall lack of standardization regarding methods for cell sorting and assessment of “stemness” confirmation. Moreover, there is a highly relevant debate regarding the best method for culturing the glioma stem cells isolated from human specimens: many groups proposed the use of adherent monolayer cultures rather than nonadherent cultures, since a homogeneous exposure to growth factors, oxygen, and nutrients increases the possibility of obtaining a more homogeneous cell population [13, 14]. On the other hand, the sphere-forming assay has been used by many laboratories as “the assay” to retrospectively isolate stem cells from GBMs [15, 16] although its reliability and benefits are still under debate. It has been reported that under appropriate conditions, cultured tumor stem cells derived from primary human glioblastomas exhibit genotype, gene expression profile, and biology of their parental primary tumors [17].

Neural stem cells were originally characterized and identified by their growth as neurospheres in a minimal medium containing growth factors [15, 16], and there is a considerable body of evidence linking the ability of brain tumors to give rise to multipassaged neurosphere cultures *ex vivo* with patient clinical outcome [18].

This paper aims to approach the CSC hypothesis to tackle the main challenge of this decade: the identification of reliable markers to defeat gliomas.

## 2. How Powerful Is CD133 as a CSCs Marker in Brain Tumors?

CD133 is considered a marker of stem cells in diverse normal tissues and cancer types. Several studies demonstrated the utility of CD133 in the enrichment of populations of cells with stem-like properties, but there is also a large body of evidence narrowing down its use as a stem cell marker. In this section of the paper, we highlight the most relevant issues concerning the role of CD133 as a stem cell marker.

Since the initial discovery in human CD34-positive hematopoietic stem cells [19, 20], the expression of CD133 has been found in endothelial progenitors [21], myogenic cells [22], prostatic epithelial stem cells [23, 24], and neural stem cells [25–27]. Cells with extensive self-renewal potential and the capacity to engraft, migrate, and undergo neural and glial differentiation after orthotopic transplantation in mice were isolated from human fetal brain and postmortem adult brain tissues using FACS sorting with CD133 antibodies. Indeed, evidence for the existence of CD133-expressing cancer stem cell populations (clonogenic expansion *in vitro* and tumor-initiating capacity *in vivo*) has been provided in numerous tumor types including leukemia [28], prostate cancer [29], colon cancer [30], lung cancer [31], hepatocellular carcinoma [32], ependymoma [33], melanoma [34], ovarian cancer [35], medulloblastoma, and glioblastoma [2, 36]. With regard to brain tumors, Singh et al. [2, 36] were the first to describe a CD133-positive tumor cell population, with stem cells characteristics that are capable of self-renewal and exact recapitulation of the original tumor when transplanted into immunodeficient mouse brains. They demonstrated that injection of as few as 100 CD133-positive cells produced a tumor, whereas injection of 100.000 CD133-negative cells did not.

Quantitative analysis of CD133-positive cells by flow cytometry has generally found them to be present at low and sometimes barely detectable levels in human gliomas, glioma sphere cultures, and established glioma cell lines [2, 37–40], consistent with the assumption that CSCs are a rare cell population in solid tumors. However, some studies have reported exceptionally high CD133-positive (20%–60%) fractions in some human GBMs and/or glioma cell lines [2, 38, 41], according to immunohistochemical findings demonstrating that many GBMs contain more than 25% CD133-positive cells [42].

Studies investigating the distribution and the prognostic value of CD133 have reported inconsistent findings [42–49]. On one hand, a quantitative correlation of glioma grade with the presence of CD133-positive cells within tumors and a negative association between CD133 expression and patient survival have been demonstrated in large cohorts of glioma patients. Opposite results were obtained by Christensen et al. [44] demonstrating no correlation between the presence of tumor cells expressing CD133 and both tumor grade and clinical outcome.

It is noteworthy that CD133 antigen has been used to enrich for cancer stem cells using flow cytometry, but whether CD133 expression measured by mRNA and/or protein on immunoblotting or immunofluorescence identifies cancer stem cells is not clearly established. However, the association of CD133 mRNA and protein with poor prognosis has been reported in several studies [50–52], including a very recent one that analyses the prognostic impact of CD133 mRNA in 48 glioblastomas [50].

A great source of inconsistency in experimental results may derive from the use of alternative antibodies recognizing different CD133 protein epitopes. The most widely used antibodies in CD133-related experiments are the CD133/1, directed against the AC133 epitope, and the CD133/2, directed against AC141 epitope. The AC133 and AC141 epitopes are both glycosylated and have distinct spatial locations [19, 20], but their molecular nature and their locations on the CD133 protein have not been determined. Several reports have documented overlap of the AC133- and AC141-positive cell populations [19, 53, 54], although currently there are no specific studies on the comparability of antibodies recognizing these two different epitopes. Indeed, immunohistochemical staining of AC133 and AC141 epitopes poses a special challenge leading some researchers to use alternative antibodies recognizing the CD133 protein. A recent study demonstrated the inconsistent CD133 detection when using different primary CD133 antibody clones in immunohistochemistry [55, 56].

In addition, the glycosylated nature of the AC133 epitope has been questioned/discussed since CD133/1 antibody can effectively detect bacterially expressed, unglycosylated CD133 [57].

Moreover, the exclusive detection of glycosylation-dependent epitopes does not exclude the expression of differentially or nonglycosylated CD133. Several studies have demonstrated that the AC133 and AC141 epitopes can be downregulated independently on the CD133 mRNA [53, 54] and that the tissue distribution of CD133 mRNA is more widespread than expression of the AC133 epitope. CD133 is expressed in differentiated epithelial cells in a variety of tissues, though it has been used to identify normal stem cells and cancer stem cells in many of these organs [58].

There are several lines of evidence to suggest the existence of CD133-negative glioma stem cells. First, CD133 is not detectable in many fresh GBM specimens [38, 41, 59] and in established glioma cell lines, which can nonetheless form tumors *in vivo* [40, 41]. Second, cells with stem cell characteristics and tumorigenic potential can be isolated from CD133-negative gliomas as well as from CD133-positive tumors. Stem cells isolated from CD133-positive and negative tumors may differ in terms of other phenotypic features, such as proliferation, invasiveness, and expression profiles. A recent study based on gene expression profile analysis of CSC isolated from CD133-positive and CD133-negative gliomas, has led to the definition of two different types of glioma stem cells: type 1 CSCs that are CD133 positive and grow as floating neurospheres and type 2 CSCs which are CD133 negative and grow adherently. Interestingly, type 1 cells were reminiscent of fetal neural stem cells and type 2 cells genetically resemble adult neural stem cells [60]. Third, both CD133-positive and CD133-negative cells isolated from the same tumor specimen can be cultured as neurospheres under serum-free conditions, and both populations of cells are able to self-renew and to initiate and propagate tumors upon xenotransplantation. Indeed, CD133-negative cells are able to generate CD133-positive progeny *in vitro* and *in vivo* [40, 61]. Chen et al. [61] recently demonstrated the existence of three different but coexisting types of glioma stem cells, which differ in CD133 expression: type 1 (CD133-negative cells able to generate CD133-positive progeny), type 2 (CD133-positive cells able to generate CD133-negative cells), and type 3 (CD133-negative which generate only CD133-negative progeny). A hierarchy lineage has been established between these three types of cells, suggesting that it is a primordial CD133-negative cell that gives rise to CD133-positive cells in some tumors.

Another important issue that needs to be considered in the criticism of the role of CD133 as marker of stem cells is its still unknown biological function.

## 3. Other Putative Markers of CSCs

Due in large part to conflicting results and irreproducibility of experiments, a lot of disagreement exists regarding the use of a specific marker or a combination of different markers to identify and isolate GBM CSCs. Besides that, there is an additional complication: as shown by Quintana and colleagues in melanoma [62, 63], many markers expressed on CSCs capable of distinguishing marker-positive and marker-negative populations were also able to regenerate the original expression pattern. The problem of the dynamicity of marker expression oscillating in a cell-cycle-dependent manner or becoming reexpressed after purification is remarkable. An emerging complication for the definition of therapeutic-suitable markers is the oscillation between the quiescent and activated states that cells undergo as well as the reversion back to a more primitive state of committed progenitors.

The currently used surface proteins used for identifying CSCs have not been shown to be necessary nor sufficient to confer stem-cell-like properties.

## 4. L1CAM

The neuronal cell adhesion molecule L1CAM (L1, CD171) is required for maintaining the growth and survival of CD133-positive glioma cells with stem-like properties [64].

It has been shown that L1CAM regulates neural cell growth, survival, and migration during central nervous system development [65], but its role in the normal adult nervous system is not well defined. In cancers, it is overexpressed in gliomas, in which it plays a role in tumor invasion [66, 67] and other solid tumors [68–70], including colorectal cancer where L1CAM functions as a prognostic indicator [71].

In gliomas, L1CAM-positive and CD133-positive cells cosegregate, and levels of L1CAM are higher in glioma cells expressing CD133 than in normal neural progenitors. However, L1CAM has not been used to identify and isolate cancer stem cells. The self-renewal capacity and the tumorigenic potential of L1CAM-positive and L1CAM-negative subpopulations of glioma cells (even together with CD133) have not been characterized yet.

Targeting L1CAM using lentiviral-mediated short hairpin RNA (shRNA) interference in CD133-positive glioma cells inhibits growth and neurosphere formation of glioma stem cells and induces their apoptosis, by regulating Olig2 expression with associated changes in the downstream effectors, p21^WAF1/CIP1 ^ [64]. Indeed, L1CAM-mediated signaling confers radioresistance in glioma stem cells by enhancing Mre11, Rad50, and Nbs1 (MRN) complex function through Myc-NBS1-ATM axis and leading to DNA checkpoint activation and DNA repair [72].

Therefore, L1CAM is an attractive therapeutic target for GBM therapy as it maintains GBM stem cells and regulates their radioresistance.

Furthermore, it has not been determined whether L1CAM itself may have a prognostic value in gliomas and may be useful in immunohistochemical studies.

## 5. CD44 and Id1

Several reports have shown the utility of the cell surface marker CD44 in the identification of cancer stem cells in different type of tumors such as breast cancer [4, 73], pancreas, and prostate carcinomas [74, 75]. There is only one example of the use of CD44 as a stem cell marker in glioblastoma [76]. Anido et al. show that CD44^high^/Id1^high^ cells are localized in the endothelial niches of the tumor tissue and possesses stem cell characteristics. Remarkably, TFG-*β* can regulate this population of cells, causing their depletion and preventing tumor initiation and recurrence. Moreover, it is shown that the high expression levels of both CD44 and Id1 are inversely correlated with survival, conferring poor prognosis in GBM patients.

A very recent paper [77] is providing new insights on the role of Id1, a transcriptional regulator of self-renewal in neural stem cells: high levels of Id1 could identify glioma stem cells. The same group has previously identified Id1 as a marker of pure stem cells since it has the tendency to reduce its expression upon differentiation [78]. Immunostaining and RT-PCR analysis confirmed higher Id1 expression in cells expressing stem-associated markers compared to cells expressing progenitors-associated markers.

Employing a PDGF- and KRAS-driven murine models of glioma, this group demonstrated that in these conditions Id1^high^ cells (represented by <1% of the total of tumor cells) are characterized by high self-renewal capacity showed by an increased percentage of cells able to form neurospheres and giving rise to heterogeneous tumors *in vivo*. The authors, while making a tight correlation between Id1 expression and stemness, are disconnecting self-renewal capacity from tumorigenic potential, since Id1^low^ expressing cells show a decreased sphere-forming capacity *in vitro* but can extensively expand *in vivo* following transplantation.

## 6. CD15

Searching for alternative enrichment markers for stem cells in brain tumors, several groups have identified CD15 as a cell surface protein selectively expressed in cells with tumor initiation capacity. CD15 (also known as SSEA-1 [stage-specific embryonic antigen-1] or LeX) is a fucose-containing trisaccharide expressed in adult neural stem/progenitor cells [79] and embryonic stem cells during neural development [80].

It has been demonstrated that in a mouse model of medulloblastoma, the Patched mutant mouse, as well as a subset of human tumors, a distinct subpopulation of cells expressing CD15 is able to propagate tumors. In those tumors, CD133 was not found to be expressed exclusively within the stem cell compartment [81].

In human glioblastomas, Son et al. have found that CD15 is an enrichment marker of stem cells in CD133-negative tumors [41]. Approximately 40% of the freshly isolated GBM specimens that they analyzed do not contain CD133-positive cells. The selection of CD15-positive cells from CD133-negative tumors enriches for cells able to form neurospheres and colonies in soft agar, to differentiate into cells expressing glial and neuronal markers, and to be highly tumorigenic *in vivo* when serially transplanted in immunocompromised mice. Furthermore, a hierarchical lineage has been established between CD15-positive and -negative cells, since the CD15-positive cells have the exclusive capacity to generate the cell heterogeneity of the primary tumor.

Even so, as in the case for CD133, CD15 does not enrich for a population of glioblastoma stem cells in every GBM tumor, and the levels of CD15-positive cells vary greatly among different brain tumor specimens.

## 7. Integrin *α*6

Targeting integrin *α*6 in GBM cells inhibits self-renewal, proliferation, and tumor formation capacity, adding insights to the identification of glioma stem cells. Integrin *α*6, important for the interaction with laminin-expressing endothelial cells in the microenvironment, is a component of the extracellular matrix whose contact is important for glioma stem cells maintenance. In the brain, laminin and integrin *α*6 regulate neural stem cell growth [82] and CSC maintenance [83]. The integrin *α*6-laminin interaction was recently reported to play an important role in the subventricular zone (SVZ) of the lateral ventricles in the adult brain [84]. Biopsy samples from glioblastoma patients showed that integrin-*α*6-positive cells are localized in close proximity to the tumor vasculature and often coexpressed the stem cell markers CD133 and nestin [85]. FACS sorting for integrin *α*6 alone or in combination with CD133 led to enrichment of cells with higher self-renewal capacity *in vitro*. Moreover, combining CD133 and integrin *α*6 expression resulted in a higher enrichment of glioma stem cells than CD133 expression alone. Xenotransplantation of integrin-*α*6-positive cells in the brains of immunocompromised mice resulted in a higher incidence of secondary tumor formation and a reduced survival than what was obtained with integrin *α*6 negative cells. Furthermore, integrin *α*6 depletion using short hairpin RNA or treatment with integrin-blocking antibody reduced both sphere growth *in vitro* and tumor formation *in vivo* [85]. In addition to advancing our ability to identify CSCs in gliomas, the findings also point to the potential of targeting integrin *α*6 for antiglioblastoma therapy.

## 8. The Stem Cell Niche as a Potential Marker's Source

So far, the vast majority of the studies regarding cancer stem cells have focused on the intrinsic properties of these cells; however, it is recognized that normal stem cells of various tissues are tightly regulated and sheltered from genotoxic insults, by the microenvironment or stem cell niche. Similarly to normal neural stem cells, the CSCs seem to have potent angiogenic properties and can recruit vessels during tumorigenesis. Moreover, it has been reported that the number of capillaries correlates with the GBM patients' prognosis [86]. Compelling data enhance the idea that a potential marker source may be the stem cell niche: there is, in fact, the possibility to find critical marker components in the cell adhesion molecules since stem cells adherent to the niche are less easily digested during isolation. This idea was born from the initial observation [87] of the preferential distribution of glioma stem cells in the perivascular area (aberrant tumor vasculature). CD133+/Nestin+ cells isolated from glioblastoma, medulloblastoma, ependymomas, and oligodendrogliomas migrate to and interact tightly with the vascular tubes formed by endothelial cells. Furthermore, cotransplanting tumor stem cells and endothelial cells into immunocompromised mice, it was shown that endothelial-derived factors (such as VEGF) accelerate the initiation and the growth of brain tumors [87]. All these observations are in addition to the fact that the niche has the ability to dedifferentiate nontumorigenic cells into tumorigenic CSCs [88, 89]. Hypoxia is a functional component of stem cell niche, promoting the maintenance of an undifferentiated cell state in normal tissues and in tumors. In hypoxic conditions, nonstem glioma cells acquire self-renewal and long-term proliferative potential, in addition to the expression of genes related to stem cell functions. The tumor cell plasticity induced by hypoxia is mainly regulated by hypoxia-inducible factors (HIFs). Differential mRNA and protein expression of HIF1*α* and HIF2*α* between nonstem and cancer stem cells have been demonstrated, although both factors have an essential role in promoting tumorigenesis. While HIF1*α* is expressed in all neoplastic cells and neural progenitors, HIF2*α* has a more specific function in cancer stem cells with no expression in normal progenitors. HIFs are critical to cancer stem cell maintenance and angiogenic drive, and expression of HIF2*α* is significantly associated with poor glioma patient survival. This implies that targeting only cancer stem cells may be insufficient to improve patients' outcomes because the nonstem cells may acquire stem cell characteristics due to effects of the microenvironment. Thus, the malignant microenvironment might be targeted by antiangiogenic therapies that would function via the disruption of stem cell maintenance.

Further insights are provided by the observation that glioblastoma stem-like cell progeny may not be confined to the neural lineage. De Maria's group demonstrated that a proportion of endothelial cells contributing to blood vessels in GBM originates from the tumor itself, directly differentiating from the tumor stem-like cells as a set of endothelial cells lining tumor vessels carry genetic abnormalities found in the tumor cells themselves [7]. Another interesting study [90] showed that the differentiation of tumor stem-like cells into endothelial cells might be mediated initially by the Notch pathway for the differentiation in endothelial progenitor cells and subsequently by the vascular-endothelium-growth-factor- (VEGF-) signaling pathway, selectively affecting the differentiation of endothelial progenitors to tumor-derived endothelial cells.

The connection between neural stem cells and the endothelial compartment seems to be critical in glioblastoma, where cancer stem cells closely interact with the vascular niche and promote angiogenesis.

## 9. Side Population

Some stem cells may additionally express ABC transporters, responsible for multidrug resistance. ATP binding (ABC) cassette transporters, able to pump the fluorescent dye HOECHST-33342 out of the cell [91], identify unlabelled “side population” (SP) highly enriched in stem cells in many tissues, including neural [92, 93]. The capacity to eject the dye HOECHST-33342 is based on ABCG2 expression, a multidrug resistance protein that is expressed in stem cells but not in downstream progenitors and thus defines an SP population highly enriched in stem cells in various tissues [94]. A growing body of evidence suggests that GBM and gliomas in general arise from the “side population” subset of cells. The SP fraction isolated from the C6 glioma cell line exhibited the stem cell properties of self-renewal and multipotency and could reform more differentiated SP-negative cells characteristic of the original cell line. These SP-positive cells were also tumorigenic *in vivo*, whereas SP-negative cells were not [95]. In a mouse model of glioma, it has been demonstrated that CSCs are enriched in the SP [96]. This cell fraction isolated from different primary tumor cells is able to sustain expansion *ex-vivo *and to generate SP and non-SP progeny [97]. The SP isolated from brain tumors is capable of neurospheres formation with self-renewal and differentiation potential, is chemoresistant, and expresses high levels of drug-transporters proteins such as MDR-1, MRP-1, and ABCG2 [98]. However, there are conflicting data showing that either the sorted SP or non-SP cells were similarly clonogenic *in vitro* and equally tumorigenic *in vivo*. In addition, when culturing SP and non-SP cells, it has been demonstrated in gastrointestinal cancer cell lines that the two populations are interconvertible, each giving rise to the other [99], even though they represent phenotypically different populations.

Several questions need to be answered regarding the role of ABCG2 in glioma biology. The Holland group has shed light on the ABCG2 activity and the resulting SP phenotype showing that ABCG2 function and localization to the plasma membrane are regulated by the PI3K and Akt pathways. Moreover, ABC transporter's function is lost in glioma endothelial cells, correlating with the blood brain barrier loss of integrity seen in glioma patients [100].

The same group previously investigated the role of nitric oxide (NO) activity in the perivascular niche using a mouse genetic model of PDGF-induced gliomas [101] and found that eNOS (endothelial nitric oxide synthase) expression is elevated in the tumor vasculature adjacent to the glioma cells, and the Notch signaling driven by NO/cGMP pathway induces the SP phenotype in primary glioma cell cultures. The production of nitric oxide was shown to increase neurosphere-forming capacity and the *in vivo* tumorigenic capacity of PDGF-driven glioma primary cultures, while its suppression prolongs mice survival. Even if the contribution of aberrant NO signaling within the niche is obviously not the only contribution to gliomagenesis, its role in tumor angiogenesis, and the involvement of the perivascular niche in the disease process, it is worthy of further investigation as a potential therapeutic target.

## 10. High ALDH Activity as a Functional Marker to Isolate CSCs

A complementary strategy for the functional identification of normal stem cells and their malignant counterparts involves the measurement of aldehyde dehydrogenase (ALDHs) activity. Aldehyde dehydrogenases (ALDH) are a family of enzymes that efficiently detoxify aldehydic products generated by reactive oxygen species and might therefore participate in cell survival. ALDH enzymes activity is important for drug resistance, cell proliferation, differentiation, and the response to oxidative stress [102–104]. High ALDH activity has been used to identify and select stem-like subsets in hematopoietic cells [105], endothelial progenitor cells, and mesenchymal and epithelial stem cells [106–108]. It is becoming increasingly clear that ALDH activity can be used, either alone or in combination with cell surface markers, to identify CSCs in hematologic malignancies and various solid carcinomas such as colon, breast, and lung [102, 103, 109, 110]. A group of investigators adopted the complex fluorescence-activated cell sorting based on high ALDH enzyme activity to select tumor-initiating cells, which correlates with enhanced clonogenicity and invasiveness *in vitro* [111, 112]. This isolation approach might present advantages: whilst the surface phenotype of a stem cell may remain stable over time, the functional activity may decline.

Moreover, it has been shown that ALDH1 inhibition induces differentiation *in vitro* and reduces clonogenicity [113]. Consequently, ALDH1 activity might be a functional correlate of an undifferentiated state of glioma cells capable of growing in neurospheres and appears to confer specific advantage to stem cells despite the fact that the molecular nature of this advantage is not yet clear. Therefore, detection of ALDH activity as a purification strategy might render the identification more reliable and with a high level of experimental reproducibility.

## 11. Cell Migration Ability as a Potential CSCs Isolation Method

We should also consider the hypothesis [114] to isolate and enrich cancer stem cell based on the heterogeneity of invasiveness of tumoral cells, according to the findings that the CSCs possess more infiltrative capability than their progeny [115].

## 12. Dye-Based Marker-Independent Method to Segregate CSCs

An alternative approach to investigate tumor-initiating potential in gliomas exploits intrinsic autofluorescence properties and distinctive morphology of human glioma cells [116]. This method can discern a subpopulation of human cells displaying autofluorescence around 520 nm upon laser excitation at 488 nm. Moreover, the same fraction retains spherogenic potential for at least 5 passages (yielding spheres with bigger size), while nonfluorescent cells lose their clonogenic capacity between passages 3 and 4. In addition to their enhanced self-renewal and multipotency, these auto-fluorescent cells also are highly tumorigenic as injection of as few as 3000 cells per mouse consistently yielded tumors. The molecular basis of this autofluorescence is unknown, but it probably reflects higher metabolic and proliferative activity.

The identification of a dye-retaining brain tumor population enables the identification of a subpopulation displaying the hallmarks of a tumor-initiating subpopulation. Several groups have recently shown that the isolation of a label-retaining cell fraction bearing robustly identifies cancer stem cells from solid tumors such as breast [117], skin [63], and pancreatic [118] cancers. To determine if such population exists in GBM as well, the Vescovi and Reynolds' groups [119] exploited the properties of CFSE (the prodrug carboxyfluorescein diacetate succinimidyl ester), which is converted by cellular esterase activity into a fluorescent compound covalently bound to proteins and retained within the cells. Since the dye is equally divided between daughter cells after division, it is possible to follow label retention and the decreasing fluorescence intensity with time. The authors observed that bright cells dilute the dye significantly slower than the overall population, exhibiting a lower frequency of cell division, and that these cells constitute a label-retaining population of glioma-initiating cells. Moreover, this cell fraction carries a greater tumor-initiation ability *in vivo* and displays migratory and infiltration capability. These observations are in agreement with the hypothesis that cancer stem cells are a slow-cycling, infrequent population endowed with the self-renewal and multipotent differentiation features of a stem cell.

## 13. Conclusion

These results support the phenotypic diversity of tumor cells, and the cellular phenotype strongly correlated with stemness and tumorigenic capacity. It is conceivable that subpopulations grouped by markers expression such as CD133, CD15, L1CAM, or integrin *α*6, for instance, represent distinct functional entities that contribute to the phenotypes of human GBMs which thus far cannot be encompassed by a single CSC marker. Considering the inconsistency between stem cell markers, there is a need to define CSCs using more precise functional markers, for example, by receptors for growth or chemotactic factors involved in stem cells maintenance or differentiation.

Thus, the heterogeneity observed in brain tumors may be correlated to the diversity on a cellular level, in which different subpopulations of glioma cells are dedicated towards different functional roles.

Given the complex genetic and epigenetic heterogeneity of GBMs, it is unlikely that the expression of a single marker will define CSCs in every tumors; hence, a combination of markers will probably best define glioma tumor stem cells. Furthermore, the ability to molecularly determine this characteristic may permit the development of more tailored brain cancers treatment.

Although several markers show promises and good potential in early studies, the lack of reliable data (caused by a lack of standardized approaches regarding methods for GBM-derived cells isolation and the procedure adopted for defining “stemness”) makes their clinical value difficult to determine.
